# Supplementary material for: Evidence of unexplained discrepancies between planned and conducted statistical analyses: a review of randomised trials
Source: BMC Med. 2020 May 29;18:137. doi: 10.1186/s12916-020-01590-1 (PMC7257229; doi:10.1186/s12916-020-01590-1)
Supplement: Supplementary file 2 — Additional file 2. Supplementary material – contains additional methods and results. [file 12916_2020_1590_MOESM2_ESM.docx]

# **Supplementary material**

**Contents**

[Supplementary material 1](#_Toc35455045)

[Appendix 1: Search strategy 2](#_Toc35455046)

[Appendix 2: Eligible trials included in the review 3](#_Toc35455047)

[Appendix 3: Additional results 11](#_Toc35455048)

[Appendix Table 1. Protocol and SAP availability by journal 11](#_Toc35455049)

[Appendix Table 2. Protocol dates (n=90) by journal 11](#_Toc35455050)

[Appendix Table 3. SAP dates (n=46) by journal 12](#_Toc35455051)

[Appendix Table 4. Comparisons of specified versus conducted statistical analysis approach – explained discrepancies 12](#_Toc35455052)

[Appendix Table 5. Comparisons of specified versus conducted statistical analysis approach by analysis element – explained discrepancies 13](#_Toc35455053)

[Appendix Table 6. Description of explained changes and additions 14](#_Toc35455054)

[Appendix Table 7. Subgroup analysis by funding as included some for profit/no for profit funding 15](#_Toc35455055)

[Appendix Table 8. Subgroup analysis by type of intervention 16](#_Toc35455056)

[Appendix Table 9. Post-hoc subgroup analysis by availability of original analysis plan pre-results 17](#_Toc35455057)

[Appendix Table 10. Post-hoc subgroup analysis by SAP availability 18](#_Toc35455058)

[Appendix Table 11. P-values by main outcomes 19](#_Toc35455059)

[Appendix Figure 1. Distribution of p-value for primary outcome by availability of pre-specified analysis approach 21](#_Toc35455060)

[Appendix Figure 2. Distribution of p-value for primary outcome by unexplained discrepancies 22](#_Toc35455061)

[Appendix Figure 3. Distribution of p-value for primary outcome for p<0.15 by unexplained discrepancies 23](#_Toc35455062)

[Appendix 4: Statistical methods and results for analysis of trial outcomes 24](#_Toc35455063)

[Appendix Table 12. Associations with p-value for primary outcome 26](#_Toc35455064)

## **Appendix 1: Search strategy**

**PubMed:**

1. “randomized controlled trial"[Publication Type] OR "random allocation" OR random*[Title/Abstract]
2. "BMJ"[Journal] OR "JAMA"[Journal] OR "Lancet (London, England)"[Journal] OR "The New England journal of medicine"[Journal] OR "PLoS medicine"[Journal] OR "Annals of internal medicine"[Journal]
3. "2018/01/01"[Date - Publication] : "2018/04/30"[Date - Publication]
4. 1 AND 2 AND 3

## **Appendix 2: Eligible trials included in the review**

Eligible Trials (n=101)

1. Blumberger DM, Vila-Rodriguez F, Thorpe KE, Feffer K, Noda Y, Giacobbe P, Knyahnytska Y, Kennedy SH, Lam RW, Daskalakis ZJ, Downar J, Effectiveness of theta burst versus high-frequency repetitive transcranial magnetic stimulation in patients with depression (THREE-D): a randomised non-inferiority trial. Lancet. 2018;391(10131):1683-92. doi: 10.016/S0140-6736(18)30295-2. Epub 2018 Apr 26.

2. Keenan JD, Bailey RL, West SK, et al., Azithromycin to Reduce Childhood Mortality in Sub-Saharan Africa. N Engl J Med. 2018;378(17):1583-92. doi: 10.056/NEJMoa1715474.

3. Campbell BCV, Mitchell PJ, Churilov L, et al., Tenecteplase versus Alteplase before Thrombectomy for Ischemic Stroke. N Engl J Med. 2018;378(17):1573-82. doi: 10.056/NEJMoa1716405.

4. Gohel MS, Heatley F, Liu X, et al., A Randomized Trial of Early Endovenous Ablation in Venous Ulceration. N Engl J Med. 2018;378(22):2105-14. doi: 10.1056/NEJMoa1801214. Epub 2018 Apr 24.

5. Lipson DA, Barnhart DVM, Brealey N, et al., Once-Daily Single-Inhaler Triple versus Dual Therapy in Patients with COPD. N Engl J Med. 2018;378(18):1671-80. doi: 10.056/NEJMoa1713901. Epub 2018 Apr 18.

6. Mullany LC, van Boetzelaer EW, Gutman JR, et al., Universal versus conditional day 3 follow-up for children with non-severe unclassified fever at the community level in the Democratic Republic of the Congo: A cluster-randomized, community-based non-inferiority trial. PLoS Med. 2018;15(4):e1002552. doi: 10.1371/journal.pmed.. eCollection 2018 Apr.

7. Kallander K, Alfven T, Funk T, et al., Universal versus conditional day 3 follow-up for children with non-severe unclassified fever at the community level in Ethiopia: A cluster-randomised non-inferiority trial. PLoS Med. 2018;15(4):e1002553. doi: 10.1371/journal.pmed.. eCollection 2018 Apr.

8. Gandhu L, Rodriguez-Abreu D, Gadgeel S, Pembrolizumab plus Chemotherapy in Metastatic Non-Small-Cell Lung Cancer. N Engl J Med. 2018;378(22):2078-92. doi: 10.1056/NEJMoa1801005. Epub 2018 Apr 16.

9. Eggermont AMM, Blank CU, Mandala M, et al., Adjuvant Pembrolizumab versus Placebo in Resected Stage III Melanoma. N Engl J Med. 2018;378(19):1789-801. doi: 10.056/NEJMoa1802357. Epub 2018 Apr 15.

10. Protopopoff N, Mosha JF, Lukole E, et al., Effectiveness of a long-lasting piperonyl butoxide-treated insecticidal net and indoor residual spray interventions, separately and together, against malaria transmitted by pyrethroid-resistant mosquitoes: a cluster, randomised controlled, two-by-two factorial design trial. Lancet. 2018;391(10130):1577-88. doi: 10.016/S0140-6736(18)30427-6. Epub 2018 Apr 11.

11. ASbell PA, Maguire MG, Pistilli M, et al., n-3 Fatty Acid Supplementation for the Treatment of Dry Eye Disease. N Engl J Med. 2018;378(18):1681-90. doi: 10.056/NEJMoa1709691. Epub 2018 Apr 13.

12. Bhatnagar R, Keenan EK, Morley AJ, et al., Outpatient Talc Administration by Indwelling Pleural Catheter for Malignant Effusion. N Engl J Med. 2018;378(14):1313-22. doi: 10.056/NEJMoa1716883.

13. The West African Network for Clinical Trials of Antimalarial Drugs (WANECAM), Pyronaridine-artesunate or dihydroartemisinin-piperaquine versus current first-line therapies for repeated treatment of uncomplicated malaria: a randomised, multicentre, open-label, longitudinal, controlled, phase 3b/4 trial. Lancet. 2018;391(10128):1378-90. doi: 10.016/S0140-6736(18)30291-5. Epub 2018 Mar 29.

14. Myer L, Phillips TK, Zerbe A, et al., Integration of postpartum healthcare services for HIV-infected women and their infants in South Africa: A randomised controlled trial. PLoS Med. 2018;15(3):e1002547. doi: 10.1371/journal.pmed.. eCollection 2018 Mar.

15. Kappos L, Bar-OR A, Cree BAC, et al., Siponimod versus placebo in secondary progressive multiple sclerosis (EXPAND): a double-blind, randomised, phase 3 study. Lancet. 2018;391(10127):1263-73. doi: 10.016/S0140-6736(18)30475-6. Epub 2018 Mar 23.

16. Seymour JF, Kipps TJ, Eichhorst B, et al., Venetoclax-Rituximab in Relapsed or Refractory Chronic Lymphocytic Leukemia. N Engl J Med. 2018;378(12):1107-20. doi: 10.056/NEJMoa1713976.

17. Franklin D, Babl FE, Schlapbach LJ, et al., A Randomized Trial of High-Flow Oxygen Therapy in Infants with Bronchiolitis. N Engl J Med. 2018;378(12):1121-31. doi: 10.056/NEJMoa1714855.

18. Motzer RJ, Tannir NM, McDermott DF, et al., Nivolumab plus Ipilimumab versus Sunitinib in Advanced Renal-Cell Carcinoma. N Engl J Med. 2018;378(14):1277-90. doi: 10.056/NEJMoa1712126. Epub 2018 Mar 21.

19. Kasividvanathan V, Rannikko AS, Borghi M, et al., MRI-Targeted or Standard Biopsy for Prostate-Cancer Diagnosis. N Engl J Med. 2018;378(19):1767-77. doi: 10.056/NEJMoa1801993. Epub 2018 Mar 18.

20. Hahn JY, Song YB, Oh JH, et al., 6-month versus 12-month or longer dual antiplatelet therapy after percutaneous coronary intervention in patients with acute coronary syndrome (SMART-DATE): a randomised, open-label, non-inferiority trial. Lancet. 2018;391(10127):1274-84. doi: 10.016/S0140-6736(18)30493-8. Epub 2018 Mar 12.

21. Molloy SF, Kanyama C, Heyderman RS, et al., Antifungal Combinations for Treatment of Cryptococcal Meningitis in Africa. N Engl J Med. 2018;378(11):1004-17. doi: 10.56/NEJMoa1710922.

22. Karlsson LO, Nilsson S, Bang M, et al., A clinical decision support tool for improving adherence to guidelines on anticoagulant therapy in patients with atrial fibrillation at risk of stroke: A cluster-randomized trial in a Swedish primary care setting (the CDS-AF study). PLoS Med. 2018;15(3):e1002528. doi: 10.1371/journal.pmed.. eCollection 2018 Mar.

23. Miller DP Jr, Denizard-Thompson N, Weaver KE, et al., Effect of a Digital Health Intervention on Receipt of Colorectal Cancer Screening in Vulnerable Patients: A Randomized Controlled Trial. Ann Intern Med. 2018;168(8):550-7. doi: 10.7326/M17-2315. Epub 018 Mar 13.

24. White WB, Saag KG, Becker MA, et al., Cardiovascular Safety of Febuxostat or Allopurinol in Patients with Gout. N Engl J Med. 2018;378(13):1200-10. doi: 10.056/NEJMoa1710895. Epub 2018 Mar 12.

25. Victor RG, Lynch K, Li N, et al., A Cluster-Randomized Trial of Blood-Pressure Reduction in Black Barbershops. N Engl J Med. 2018;378(14):1291-301. doi: 10.056/NEJMoa1717250. Epub 2018 Mar 12.

26. Harrison SA, Rinella ME, Abdelmalek MF, et al., NGM282 for treatment of non-alcoholic steatohepatitis: a multicentre, randomised, double-blind, placebo-controlled, phase 2 trial. Lancet. 2018;391(10126):1174-85. doi: 10.016/S0140-6736(18)30474-4. Epub 2018 Mar 5.

27. Jourdain G, Ngo-Giang-Huong N, Harrison L, et al., Tenofovir versus Placebo to Prevent Perinatal Transmission of Hepatitis B. N Engl J Med. 2018;378(10):911-23. doi: 10.1056/NEJMoa1708131.

28. Powell-Jackson T, Fabbri C, Dutt V, et al., Effect and cost-effectiveness of educating mothers about childhood DPT vaccination on immunisation uptake, knowledge, and perceptions in Uttar Pradesh, India: A randomised controlled trial. PLoS Med. 2018;15(3):e1002519. doi: 10.1371/journal.pmed.. eCollection 2018 Mar.

29. McKeever T, Mortimer K, Wilson A, et al., Quadrupling Inhaled Glucocorticoid Dose to Abort Asthma Exacerbations. N Engl J Med. 2018;378(10):902-10. doi: 10.1056/NEJMoa1714257. Epub 2018 Mar 3.

30. Jackson DJ, Bacharier LB, Mauger DT, et al., Quintupling Inhaled Glucocorticoids to Prevent Childhood Asthma Exacerbations. N Engl J Med. 2018;378(10):891-901. doi: 10.1056/NEJMoa1710988. Epub 2018 Mar 3.

31. McManus RJ, Mant J, Franssen, et al., Efficacy of self-monitored blood pressure, with or without telemonitoring, for titration of antihypertensive medication (TASMINH4): an unmasked randomised controlled trial. Lancet. 2018;391(10124):949-59. doi: 10.1016/S0140-6736(18)30309-X. Epub 2018 Feb 27.

32. Annane D, Renault A, Brun-Buisson C, et al., Hydrocortisone plus Fludrocortisone for Adults with Septic Shock. N Engl J Med. 2018;378(9):809-18. doi: 10.1056/NEJMoa1705716.

33. Self WH, Semler MW, Wanderer JP, et al., Balanced Crystalloids versus Saline in Noncritically Ill Adults. N Engl J Med. 2018;378(9):819-28. doi: 10.1056/NEJMoa1711586. Epub 2018 Feb 27.

34. Semler MW, Self WH, Wanderer JP, et al., Balanced Crystalloids versus Saline in Critically Ill Adults. N Engl J Med. 2018;378(9):829-39. doi: 10.1056/NEJMoa1711584. Epub 2018 Feb 27.

35. Thorn BE, Eyer JC, Van Dyke BP, et al., Literacy-Adapted Cognitive Behavioral Therapy Versus Education for Chronic Pain at Low-Income Clinics: A Randomized Controlled Trial. Ann Intern Med. 2018;168(7):471-80. doi: 10.7326/M17-0972. Epub 2018 Feb 27.

36. Bines JE, Thobari JA, Satria CD, et al., Human Neonatal Rotavirus Vaccine (RV3-BB) to Target Rotavirus from Birth. N Engl J Med. 2018;378(8):719-30. doi: 10.1056/NEJMoa1706804.

37. Anderson DR, Dunbar M, Murnaghan J, et al., Aspirin or Rivaroxaban for VTE Prophylaxis after Hip or Knee Arthroplasty. N Engl J Med. 2018;378(8):699-707. doi: 10.1056/NEJMoa1712746.

38. Kingsbury SR, Tharmanathan P, Keding A, et al., Hydroxychloroquine Effectiveness in Reducing Symptoms of Hand Osteoarthritis: A Randomized Trial. Ann Intern Med. 2018;168(6):385-95. doi: 10.7326/M17-1430. Epub 2018 Feb 20.

39. Kaufmann R, Halm JA, Eker HH, et al., Mesh versus suture repair of umbilical hernia in adults: a randomised, double-blind, controlled, multicentre trial. Lancet. 2018;391(10123):860-9. doi: 10.1016/S0140-6736(18)30298-8. Epub 2018 Feb 17.

40. Heinemann L, Freckmann G, Ehmann D, et al., Real-time continuous glucose monitoring in adults with type 1 diabetes and impaired hypoglycaemia awareness or severe hypoglycaemia treated with multiple daily insulin injections (HypoDE): a multicentre, randomised controlled trial. Lancet. 2018;391(10128):1367-77. doi: 10.016/S0140-6736(18)30297-6. Epub 2018 Feb 16.

41. Mercuri, E, Darras BT, Chiriboga CA, et al., Nusinersen versus Sham Control in Later-Onset Spinal Muscular Atrophy. N Engl J Med. 2018;378(7):625-35. doi: 10.1056/NEJMoa1710504.

42. Kudo M, Finn RS, Qin S, et al., Lenvatinib versus sorafenib in first-line treatment of patients with unresectable hepatocellular carcinoma: a randomised phase 3 non-inferiority trial. Lancet. 2018;391(10126):1163-73. doi: 10.016/S0140-6736(18)30207-1.

43. Papi A, Vestbo J, Fabbri L, et al., Extrafine inhaled triple therapy versus dual bronchodilator therapy in chronic obstructive pulmonary disease (TRIBUTE): a double-blind, parallel group, randomised controlled trial. Lancet. 2018;391(10125):1076-84. doi: 10.16/S0140-6736(18)30206-X. Epub 2018 Feb 9.

44. Smith MR, Saad F, Chowdhury S, et al., Apalutamide Treatment and Metastasis-free Survival in Prostate Cancer. N Engl J Med. 2018;378(15):1408-18. doi: 10.056/NEJMoa1715546. Epub 2018 Feb 8.

45. Raskind MA, Peskind ER, Chow B, et al., Trial of Prazosin for Post-Traumatic Stress Disorder in Military Veterans. N Engl J Med. 2018;378(6):507-17. doi: 10.1056/NEJMoa1507598.

46. Ballard C, Corbett A, Orrell M, et al., Impact of person-centred care training and person-centred activities on quality of life, agitation, and antipsychotic use in people with dementia living in nursing homes: A cluster-randomised controlled trial. PLoS Med. 2018;15(2):e1002500. doi: 10.1371/journal.pmed.. eCollection 2018 Feb.

47. Thiele EA, Marsh ED, French JA, et al., Cannabidiol in patients with seizures associated with Lennox-Gastaut syndrome (GWPCARE4): a randomised, double-blind, placebo-controlled phase 3 trial. Lancet. 2018;391(10125):1085-96. doi: 10.16/S0140-6736(18)30136-3. Epub 2018 Jan 26.

48. Marrouche NF, Brachmann J, Andresen D, et al., Catheter Ablation for Atrial Fibrillation with Heart Failure. N Engl J Med. 2018;378(5):417-27. doi: 10.1056/NEJMoa1707855.

49. Honig LS, Vellas B, Woodward M, et al., Trial of Solanezumab for Mild Dementia Due to Alzheimer's Disease. N Engl J Med. 2018;378(4):321-30. doi: 10.1056/NEJMoa1705971.

50. Albers GW, Marks MP, Kemp S, et al., Thrombectomy for Stroke at 6 to 16 Hours with Selection by Perfusion Imaging. N Engl J Med. 2018;378(8):708-18. doi: 10.1056/NEJMoa1713973. Epub 2018 Jan 24.

51. Opoku NO, Bakajika DK, Kanza EM, et al., Single dose moxidectin versus ivermectin for Onchocerca volvulus infection in Ghana, Liberia, and the Democratic Republic of the Congo: a randomised, controlled, double-blind phase 3 trial. Lancet. 2018;17(17):32844-1.

52. Venkatesh B, Finfer S, Cohen J, et al., Adjunctive Glucocorticoid Therapy in Patients with Septic Shock. N Engl J Med. 2018;378(9):797-808. doi: 10.1056/NEJMoa1705835. Epub 2018 Jan 19.

53. Van Driel WJ, Koole SN, Sikorska K, et al., Hyperthermic Intraperitoneal Chemotherapy in Ovarian Cancer. N Engl J Med. 2018;378(3):230-40. doi: 10.1056/NEJMoa1708618.

54. Fox GJ, Nhung NV, Sy DN, et al., Household-Contact Investigation for Detection of Tuberculosis in Vietnam. N Engl J Med. 2018;378(3):221-9. doi: 10.1056/NEJMoa1700209.

55. Vuong LN, Dang VQ, Ho TM, et al., IVF Transfer of Fresh or Frozen Embryos in Women without Polycystic Ovaries. N Engl J Med. 2018;378(2):137-47. doi: 10.1056/NEJMoa1703768.

56. Shi Y, Sun Y, Hao C, et al., Transfer of Fresh versus Frozen Embryos in Ovulatory Women. N Engl J Med. 2018;378(2):126-36. doi: 10.1056/NEJMoa1705334.

57. Sullivan KM, Goldmuntz EA, Keyes-Elstein L, et al., Myeloablative Autologous Stem-Cell Transplantation for Severe Scleroderma. N Engl J Med. 2018;378(1):35-47.

58. Bath PM, Woodhouse LJ, Appleton JP, et al., Antiplatelet therapy with aspirin, clopidogrel, and dipyridamole versus clopidogrel alone or aspirin and dipyridamole in patients with acute cerebral ischaemia (TARDIS): a randomised, open-label, phase 3 superiority trial. Lancet. 2018;391(10123):850-9. doi: 10.1016/S0140-6736(17)32849-0. Epub 2017 Dec 20.

59. Weiss NS, Nahuis MJ, Bordewijk E, et al., Gonadotrophins versus clomifene citrate with or without intrauterine insemination in women with normogonadotropic anovulation and clomifene failure (M-OVIN): a randomised, two-by-two factorial trial. Lancet. 2018;391(10122):758-65. doi: 10.1016/S0140-6736(17)33308-1. Epub 2017 Dec 19.

60. Powles T, Duran I, van der Heijden MS, et al., Atezolizumab versus chemotherapy in patients with platinum-treated locally advanced or metastatic urothelial carcinoma (IMvigor211): a multicentre, open-label, phase 3 randomised controlled trial. Lancet. 2018;391(10122):748-57. doi: 10.1016/S0140-6736(17)33297-X. Epub 2017 Dec 18.

61. Zheng MX, Hua XY, Feng JT, et al., Trial of Contralateral Seventh Cervical Nerve Transfer for Spastic Arm Paralysis. N Engl J Med. 2018;378(1):22-34. doi: 10.1056/NEJMoa1615208. Epub 2017 Dec 20.

62. Shepstone L, Lenaghan E, Cooper C, et al., Screening in the community to reduce fractures in older women (SCOOP): a randomised controlled trial. Lancet. 2018;391(10122):741-7. doi: 10.1016/S0140-6736(17)32640-5. Epub 2017 Dec 16.

63. Thwaites GE, Scarborough M, Szubert A, et al. Adjunctive rifampicin for Staphylococcus aureus bacteraemia (ARREST): a multicentre, randomised, double-blind, placebo-controlled trial. Lancet. 2018;391(10121):668-78. doi: 10.1016/S0140-6736(17)32456-X. Epub 2017 Dec 14.

64. Mateos MV, Dimopoulos MA, Cavo M, et al., Daratumumab plus Bortezomib, Melphalan, and Prednisone for Untreated Myeloma. N Engl J Med. 2018;378(6):518-28. doi: 10.1056/NEJMoa1714678. Epub 2017 Dec 12.

65. Raskob GE, van Es N, Verhamme P, et al., Edoxaban for the Treatment of Cancer-Associated Venous Thromboembolism. N Engl J Med. 2018;378(7):615-24. doi: 10.1056/NEJMoa1711948. Epub 2017 Dec 12.

66. Connors JM, Jurczak W, Straus DJ, et al., Brentuximab Vedotin with Chemotherapy for Stage III or IV Hodgkin's Lymphoma. N Engl J Med. 2018;378(4):331-44. doi: 10.1056/NEJMoa1708984. Epub 2017 Dec 10.

67. Lean MEJ, Leslie WS, Barnes AC, et al., Primary care-led weight management for remission of type 2 diabetes (DiRECT): an open-label, cluster-randomised trial. Lancet. 2018;391(10120):541-51. doi: 10.1016/S0140-6736(17)33102-1. Epub 2017 Dec 5.

68. de Winter RJ, Katagiri Y, Asano T, et al., A sirolimus-eluting bioabsorbable polymer-coated stent (MiStent) versus an everolimus-eluting durable polymer stent (Xience) after percutaneous coronary intervention (DESSOLVE III): a randomised, single-blind, multicentre, non-inferiority, phase 3 trial. Lancet. 2018;391(10119):431-40. doi: 10.1016/S0140-6736(17)33103-3. Epub 2017 Dec 5.

69. Farquhar CM, Liu E, Armstrong S, et al., Intrauterine insemination with ovarian stimulation versus expectant management for unexplained infertility (TUI): a pragmatic, open-label, randomised, controlled, two-centre trial. Lancet. 2018;391(10119):441-50. doi: 10.1016/S0140-6736(17)32406-6. Epub 2017 Nov 23.

70. Beard DJ, Rees JL, Cook JA, et al., Arthroscopic subacromial decompression for subacromial shoulder pain (CSAW): a multicentre, pragmatic, parallel group, placebo-controlled, three-group, randomised surgical trial. Lancet. 2018;391(10118):329-38. doi: 10.1016/S0140-6736(17)32457-1. Epub 2017 Nov 20.

71. Soria JC, Ohe Yuichiro, Vansteenkiste J, et al., Osimertinib in Untreated EGFR-Mutated Advanced Non-Small-Cell Lung Cancer. N Engl J Med. 2018;378(2):113-25. doi: 10.1056/NEJMoa1713137. Epub 2017 Nov 18.

72. Lee JD, Nunes EV, Novo P, et al., Comparative effectiveness of extended-release naltrexone versus buprenorphine-naloxone for opioid relapse prevention (X:BOT): a multicentre, open-label, randomised controlled trial. Lancet. 2018;391(10118):309-18. doi: 10.1016/S0140-6736(17)32812-X. Epub 2017 Nov 14.

73. Weisbord SD, Gallagher M, Jneid H, et al., Outcomes after Angiography with Sodium Bicarbonate and Acetylcysteine. N Engl J Med. 2018;378(7):603-14. doi: 10.1056/NEJMoa1710933. Epub 2017 Nov 12.

74. Nogueira RG, Jadhav AP, HAussen DC, et al., Thrombectomy 6 to 24 Hours after Stroke with a Mismatch between Deficit and Infarct. N Engl J Med. 2018;378(1):11-21. doi: 10.1056/NEJMoa1706442. Epub 2017 Nov 11.

75. Reignier J, Boisrame-Helms J, Brisard L, et al., Enteral versus parenteral early nutrition in ventilated adults with shock: a randomised, controlled, multicentre, open-label, parallel-group study (NUTRIREA-2). Lancet. 2018;391(10116):133-43. doi: 10.1016/S0140-6736(17)32146-3. Epub 2017 Nov 8.

76. Mesu VKBK, Kalonji WM, Bardonneau et al., Oral fexinidazole for late-stage African Trypanosoma brucei gambiense trypanosomiasis: a pivotal multicentre, randomised, non-inferiority trial. Lancet. 2018;391(10116):144-54. doi: 10.1016/S0140-6736(17)32758-7. Epub 2017 Nov 4.

77. van Brunschot S, can Grinsven J, van Santvoort HC, et al., Endoscopic or surgical step-up approach for infected necrotising pancreatitis: a multicentre randomised trial. Lancet. 2018;391(10115):51-8. doi: 10.1016/S0140-6736(17)32404-2. Epub 2017 Nov 3.

78. Al-Lamee R, Thompson D, Dehbi HM, et al., Percutaneous coronary intervention in stable angina (ORBITA): a double-blind, randomised controlled trial. Lancet. 2018;391(10115):31-40. doi: 10.1016/S0140-6736(17)32714-9. Epub 2017 Nov 2.

79. Varenne O, Cook S, Sideris G, et al., Drug-eluting stents in elderly patients with coronary artery disease (SENIOR): a randomised single-blind trial. Lancet. 2018;391(10115):41-50. doi: 10.1016/S0140-6736(17)32713-7. Epub 2017 Nov 1.

80. McDermott MM, Spring B, Berger JS, et al., Effect of a Home-Based Exercise Intervention of Wearable Technology and Telephone Coaching on Walking Performance in Peripheral Artery Disease: The HONOR Randomized Clinical Trial. JAMA. 2018;319(16):1665-76. doi: 10.001/jama.2018.3275.

81. Zhao Q, Yunpeng Z, Xu Z, et al., Effect of Ticagrelor Plus Aspirin, Ticagrelor Alone, or Aspirin Alone on Saphenous Vein Graft Patency 1 Year After Coronary Artery Bypass Grafting: A Randomized Clinical Trial. JAMA. 2018;319(16):1677-86. doi: 10.001/jama.2018.3197.

82. Wang C, Schmid CH, Fielding RA, et al., Effect of tai chi versus aerobic exercise for fibromyalgia: comparative effectiveness randomized controlled trial. BMJ. 2018;360:k851.

83. Berwanger O, Santucci EV, de Barros ESPGM, et al., Effect of Loading Dose of Atorvastatin Prior to Planned Percutaneous Coronary Intervention on Major Adverse Cardiovascular Events in Acute Coronary Syndrome: The SECURE-PCI Randomized Clinical Trial. JAMA. 2018;319(13):1331-40. doi: 10.001/jama.2018.444.

84. Krebs EE, Gravely A, Nugent BA, et al., Effect of Opioid vs Nonopioid Medications on Pain-Related Function in Patients With Chronic Back Pain or Hip or Knee Osteoarthritis Pain: The SPACE Randomized Clinical Trial. JAMA. 2018;319(9):872-82. doi: 10.1001/jama.2018.0899.

85. Martin RM, Donovan JL, Turner EL, et al., Effect of a Low-Intensity PSA-Based Screening Intervention on Prostate Cancer Mortality: The CAP Randomized Clinical Trial. JAMA. 2018;319(9):883-95. doi: 10.1001/jama.2018.0154.

86. Labhardt ND, Ringera I, Lejone TI, et al., Effect of Offering Same-Day ART vs Usual Health Facility Referral During Home-Based HIV Testing on Linkage to Care and Viral Suppression Among Adults With HIV in Lesotho: The CASCADE Randomized Clinical Trial. JAMA. 2018;319(11):1103-12. doi: 10.001/jama.2018.1818.

87. Parshuram CS, Dryden-Palmer K, Farrell C, et al., Effect of a Pediatric Early Warning System on All-Cause Mortality in Hospitalized Pediatric Patients: The EPOCH Randomized Clinical Trial. JAMA. 2018;319(10):1002-12. doi: 10.1/jama.2018.0948.

88. van Meenen DMP, van der Hoeven SM, Binnekade JM, et al., Effect of On-Demand vs Routine Nebulization of Acetylcysteine With Salbutamol on Ventilator-Free Days in Intensive Care Unit Patients Receiving Invasive Ventilation: A Randomized Clinical Trial. JAMA. 2018;319(10):993-1001. doi: 10./jama.2018.0949.

89. Kaye KS, Bhowmick T, Metallidis S, et al., Effect of Meropenem-Vaborbactam vs Piperacillin-Tazobactam on Clinical Cure or Improvement and Microbial Eradication in Complicated Urinary Tract Infection: The TANGO I Randomized Clinical Trial. JAMA. 2018;319(8):788-99. doi: 10.1001/jama.2018.0438.

90. Jabre P, Penaloza A, Pinero D., et al., Effect of Bag-Mask Ventilation vs Endotracheal Intubation During Cardiopulmonary Resuscitation on Neurological Outcome After Out-of-Hospital Cardiorespiratory Arrest: A Randomized Clinical Trial. JAMA. 2018;319(8):779-87. doi: 10.1001/jama.2018.0156.

91. Gardner CD, Trepanowski JF, Del Gobbo LC, et al., Effect of Low-Fat vs Low-Carbohydrate Diet on 12-Month Weight Loss in Overweight Adults and the Association With Genotype Pattern or Insulin Secretion: The DIETFITS Randomized Clinical Trial. JAMA. 2018;319(7):667-79. doi: 10.1001/jama.2018.0245.

92. van den Boogaard M, Slooter AJC, Bruggemann RJM, et al., Effect of Haloperidol on Survival Among Critically Ill Adults With a High Risk of Delirium: The REDUCE Randomized Clinical Trial. JAMA. 2018;319(7):680-90. doi: 10.1001/jama.2018.0160.

93. Huffman MD, Mohanan RP, Devarajan R, et al., Effect of a Quality Improvement Intervention on Clinical Outcomes in Patients in India With Acute Myocardial Infarction: The ACS QUIK Randomized Clinical Trial. JAMA. 2018;319(6):567-78. doi: 10.1001/jama.2017.21906.

94. Freund Y, Cachanado M, Aubry A, et al., Effect of the Pulmonary Embolism Rule-Out Criteria on Subsequent Thromboembolic Events Among Low-Risk Emergency Department Patients: The PROPER Randomized Clinical Trial. JAMA. 2018;319(6):559-66. doi: 10.1001/jama.2017.21904.

95. Adab P, Pallan MJ, Lancashire ER, et al., Effectiveness of a childhood obesity prevention programme delivered through schools, targeting 6 and 7 year olds: cluster randomised controlled trial (WAVES study). BMJ. 2018;360:k211.

96. Boden I, Skinner EH, Browning L, et al., Preoperative physiotherapy for the prevention of respiratory complications after upper abdominal surgery: pragmatic, double blinded, multicentre randomised controlled trial. BMJ. 2018;360:j5916.

97. Foa EB, McLean CP, Zang Y, et al., Effect of Prolonged Exposure Therapy Delivered Over 2 Weeks vs 8 Weeks vs Present-Centered Therapy on PTSD Symptom Severity in Military Personnel: A Randomized Clinical Trial. JAMA. 2018;319(4):354-64. doi: 10.1001/jama.2017.21242.

98. Peterli R, Wolnerhanssen BK, Peters T, et al., Effect of Laparoscopic Sleeve Gastrectomy vs Laparoscopic Roux-en-Y Gastric Bypass on Weight Loss in Patients With Morbid Obesity: The SM-BOSS Randomized Clinical Trial. JAMA. 2018;319(3):255-65. doi: 10.1001/jama.2017.20897.

99. Salminen P, Helmio M, Ovaska J, et al., Effect of Laparoscopic Sleeve Gastrectomy vs Laparoscopic Roux-en-Y Gastric Bypass on Weight Loss at 5 Years Among Patients With Morbid Obesity: The SLEEVEPASS Randomized Clinical Trial. JAMA. 2018;319(3):241-54. doi: 10.1001/jama.2017.20313.

100. Knip M, Akerblom HK, Al Taji E, et al., Effect of Hydrolyzed Infant Formula vs Conventional Formula on Risk of Type 1 Diabetes: The TRIGR Randomized Clinical Trial. JAMA. 2018;319(1):38-48. doi: 10.1001/jama.2017.19826.

101. Feldman TE, Reardon MJ, Rajagopal V, et al., Effect of Mechanically Expanded vs Self-Expanding Transcatheter Aortic Valve Replacement on Mortality and Major Adverse Clinical Events in High-Risk Patients With Aortic Stenosis: The REPRISE III Randomized Clinical Trial. JAMA. 2018;319(1):27-37. doi: 10.1001/jama.2017.19132.

## **Appendix 3: Additional results**

### **Appendix Table 1. Protocol and SAP availability by journal**

| **Outcome** | **Annals of Internal Medicine (n=3)** | **The BMJ**  **(n=3)** | **JAMA (n=19)** | **Lancet (n=28)** | **NEJM (n=42)** | **PLOS Medicine (n=6)** | **All**  **(n=101)** |
| --- | --- | --- | --- | --- | --- | --- | --- |
| Protocol available (n, %) |  |  |  |  |  |  |  |
| No | 1 (33%) | 0 (0%) | 0 (0%) | 10 (36%) | 0 (0%) | 0 (0%) | 11 (11%) |
| Yes | 2 (67%) | 3 (100%) | 19 (100%) | 18 (65%) | 42 (100%) | 6 (100%) | 90 (89%) |
| Separate SAP available (n, %) |  |  |  |  |  |  |  |
| No | 2 (67%) | 3 (100%) | 9 (47%) | 23 (82%) | 12 (29%) | 6 (100%) | 55 (54%) |
| Yes | 1 (33%) | 0 (0%) | 10 (53%) | 5 (18%) | 30 (71%) | 0 (0%) | 46 (46%) |

### **Appendix Table 2. Protocol dates (n=90) by journal**

| **Date of earliest version of protocol** | **Annals of Internal Medicine (n=2)** | **The BMJ**  **(n=3)** | **JAMA (n=19)** | **Lancet (n=18)** | **NEJM (n=42)** | **PLOS Medicine (n=6)** | **All (n=90)** |
| --- | --- | --- | --- | --- | --- | --- | --- |
| Before recruitment began | 2 (100%) | 0 | 7 (37%) | 2 (11%) | 34 (81%) | 0 (0%) | 45 (50%) |
| During recruitment | 0 | 0 | 8 (42%) | 9 (50%) | 1 (2%) | 1 (17%) | 19 (21%) |
| After recruitment ended | 0 | 2 (67%) | 3 (16%) | 2 (11%) | 0 (0%) | 1 (17%) | 8 (9%) |
| Date unavailable | 0 | 1 (33%) | 1 (5%) | 5 (28%) | 7 (17%) | 4 (67%) | 18 (20%) |

### **Appendix Table 3. SAP dates (n=46) by journal**

| **Date of earliest version of SAP** | **Annals of Internal Medicine (n=1)** | **The BMJ**  **(n=0)** | **JAMA (n=10)** | **Lancet (n=5)** | **NEJM (n=30)** | **PLOS Medicine (n=0)** | **All (n=46)** |
| --- | --- | --- | --- | --- | --- | --- | --- |
| Before recruitment began | 0 (0%) | 0 (0%) | 2 (20%) | 0 (0%) | 7 (23%) | 0 (0%) | 9 (20%) |
| During recruitment | 0 (0%) | 0 (0%) | 2 (20%) | 1 (20%) | 10 (33%) | 0 (0%) | 13 (28%) |
| After recruitment ended | 1 (100%) | 0 (0%) | 3 (30%) | 4 (80%) | 5 (17%) | 0 (0%) | 13 (28%) |
| Date unavailable | 0 (0%) | 0 (0%) | 3 (30%) | 0 (0%) | 8 (27%) | 0 (0%) | 11 (24%) |

### **Appendix Table 4. Comparisons of specified versus conducted statistical analysis approach – explained discrepancies**

| **Outcome** | **N (%)** |
| --- | --- |
| Explained discrepancy |  |
| No discrepancies | 5 (6%) |
| ≥1 explained discrepancies^a^ | 29 (33%) |
| Only unexplained discrepancies | 42 (47%) |
| Unclear^b^ | 13 (15%) |
| Total no. of analysis elements with explained  Discrepancy |  |
| 0^c^ | 47 (53%) |
| 1 | 15 (17%) |
| 2 | 9 (10%) |
| 3 | 5 (6%) |
| 4 | 0 (0%) |
| Unclear^a^ | 13 (15%) |
| Where was discrepancy first explained^d^ |  |
| Later version protocol | 8 (9%) |
| Later version SAP | 19 (21%) |
| Trial results publication | 2 (2%) |

^a^Includes 17 trials where all discrepancies explained, and 12 trials with both explained and unexplained discrepancies. ^b^Unclear if discrepancy occurred (n=11), unclear if discrepancy explained (n=2). ^c^Includes 5 trials with no explained discrepancies and 42 trials with only unexplained discrepancies. ^d^For multiple explained changes/additions result is for last explained change/addition

### **Appendix Table 5. Comparisons of specified versus conducted statistical analysis approach by analysis element – explained discrepancies**

| **Outcome** | **Analysis population** | **Analysis model** | **Covariates** | **Missing data** |
| --- | --- | --- | --- | --- |
| No. trials with explained discrepancy | 10 (11%) | 17(19%) | 14 (16%) | 8 (9%) |
| No. trials with explained addition | 7 (8%) | 5 (6%) | 4 (4%) | 6 (7%) |
| No. trials with explained change | 3 (3%) | 12 (13%) | 10 (11%) | 2 (2%) |
| Where was discrepancy first explained |  |  |  |  |
| Later version protocol | 4 (4%) | 5 (6%) | 3 (3%) | 3 (3%) |
| Later version SAP | 5 (6%) | 11 (12%) | 11 (12%) | 5 (6%) |
| Trial results publication | 1 (1%) | 1 (1%) | 0 | 0 |

### **Appendix Table 6. Description of explained changes and additions**

| **Explained changes** | **N (%)** |
| --- | --- |
| Analysis population |  |
| Changed via specifying additional exclusions | 2 (2%) |
| Changed from subset to total randomized as results did not differ | 1 (1%) |
| Analysis model |  |
| Changed model | 12 (13%) |
| Covariates |  |
| Changed from unadjusted to adjusted | 4 (4%) |
| Changed from adjusted to unadjusted | 2 (2%) |
| Changed covariates adjusted for | 4 (4%) |
| Missing data |  |
| Changed from including only patients with available data at final time-point to including patients with available data at any time-point | 1 (1%) |
| Changed from multiple imputation to complete case | 1 (1%) |
| Explained additions |  |
| Analysis population |  |
| Not mentioned in Original Analysis Plan | 2 (2%) |
| Incomplete detail given in Original Analysis Plan | 5 (6%) |
| Analysis model |  |
| Not mentioned in Original Analysis Plan | 1 (1%) |
| Incomplete detail given in Original Analysis Plan | 4 (4%) |
| Covariates |  |
| Not mentioned in Original Analysis Plan | 2 (2%) |
| Incomplete detail given in Original Analysis Plan | 2 (2%) |
| Missing data |  |
| Not mentioned in Original Analysis Plan | 4 (4%) |
| Incomplete detail given in Original Analysis Plan | 2 (2%) |

### **Appendix Table 7. Subgroup analysis by funding - included some for profit/no for profit funding**

| **Unexplained discrepancy** | **Not for profit (N=61)** | **For profit (N=28)** | **All (N=89)** |
| --- | --- | --- | --- |
| None | 16 (26%) | 6 (21%) | 22^a^ (25%) |
| Yes | 41 (67%) | 13 (46%) | 54 (61%) |
| Unclear | 4 (7%) | 9 (32%) | 13^c^ (15%) |
| Total no. of analysis elements with unexplained discrepancy |  |  |  |
| 0 | 16 (26%) | 6 (21%) | 22^a^ (25%) |
| 1 | 18 (30%) | 7 (25%) | 25 (28%) |
| 2 | 12 (20%) | 4 (14%) | 16 (18%) |
| 3 | 10 (16%) | 1 (4%) | 11 (12%) |
| 4 | 1 (2%) | 1 (4%) | 2 (2%) |
| Unclear | 4 (7%) | 9 (32%) | 13^c^ (15%) |
| Analysis population |  |  |  |
| No. trials with unexplained discrepancy | 23 (38%) | 5 (18%) | 28 (31%) |
| No. trials with unexplained addition | 14 (23%) | 2 (7%) | 16 (18%) |
| No. trials with unexplained change | 9 (15%) | 3 (11%) | 12 (13%) |
| Analysis model |  |  |  |
| No. trials with unexplained discrepancy | 25 (41%) | 6 (21%) | 30 (34%) |
| No. trials with unexplained addition | 13 (21%) | 4^b^ (14%) | 16 (18%) |
| No. trials with unexplained change | 13 (21%) | 2^b^ (7%) | 15 (17%) |
| Covariates |  |  |  |
| No. trials with unexplained discrepancy | 16 (26%) | 7 (25%) | 23 (26%) |
| No. trials with unexplained addition | 4 (7%) | 3 (11%) | 7 (8%) |
| No. trials with unexplained change | 12 (20%) | 4 (14%) | 16 (18%) |
| Missing data |  |  |  |
| No. trials with unexplained discrepancy | 12 (20%) | 4 (14%) | 16 (18%) |
| No. trials with unexplained addition | 10 (16%) | 3 (11%) | 13 (15%) |
| No. trials with unexplained change | 2 (3%) | 1 (4%) | 3 (3%) |

^a^ Included 5 trials with no discrepancies (3 not for profit and 2 for profit) and trials 17 trials with all discrepancies explained (13 not for profit and 4 for profit). ^b^One trial has a change and an addition. ^c^Unclear if discrepancy occurred (n=11 including 3 not for profit and 8 for profit), unclear if discrepancy explained (n=2 including 1 not for profit and 1 for profit).

### **Appendix Table 8. Subgroup analysis by type of intervention**

| **Outcome** | **Pharma (n=47)** | **Surgical (n=12)** | **Psychological/ behavioural/ educational (n=9)** | **Other (n=19)** | **Multiple (n=2)** | **All (n=89)** |
| --- | --- | --- | --- | --- | --- | --- |
| Unexplained discrepancy |  |  |  |  |  |  |
| None | 13 (28%) | 2 (17%) | 0 (0%) | 6 (32%) | 1 (50%) | 22^a^ (25%) |
| Yes | 25 (53%) | 10 (83%) | 9 (100%) | 9 (47%) | 1 (50%) | 54 (61%) |
| Unclear | 9 (19%) | 0 (0%) | 0 (0%) | 4 (21%) | 0 (0%) | 13^b^ (15%) |
| Total no. of analysis elements with unexplained discrepancy |  |  |  |  |  |  |
| 0 | 13 (28%) | 2 (17%) | 0 (0%) | 6 (32%) | 1 (50%) | 22^a^ (25%) |
| 1 | 15 (32%) | 5 (42%) | 2 (22%) | 2 (11%) | 1 (50%) | 25 (28%) |
| 2 | 7 (15%) | 1 (8%) | 3 (33%) | 5 (26%) | 0 (0%) | 16 (18%) |
| 3 | 3 (6%) | 3 (25%) | 3 (33%) | 2 (11%) | 0 (0%) | 11 (12%) |
| 4 | 0 (0%) | 1 (8%) | 1 (11%) | 0 (0%) | 0 (0%) | 2 (2%) |
| Unclear | 9 (19%) | 0 (0%) | 0 (0%) | 4 (21%) | 0 (0%) | 13^b^ (15%) |
| Analysis population |  |  |  |  |  |  |
| No. trials with unexplained discrepancy | 13 (28%) | 4 (33%) | 7 (78%) | 4 (21%) | 0 (0%) | 28 (31%) |
| No. trials with unexplained addition | 6 (13%) | 3 (25%) | 5 (56%) | 2 (11%) | 0 (0%) | 16 (18%) |
| No. trials with unexplained change | 7 (15%) | 1 (8%) | 2 (22%) | 2 (11%) | 0 (0%) | 12 (13%) |
| Analysis model |  |  |  |  |  |  |
| No. trials with unexplained discrepancy | 12 (26%) | 6 (50%) | 6 (67%) | 6 (32%) | 1 (50%) | 31 (35%) |
| No. trials with unexplained addition | 7 (15%) | 4 (33%) | 5 (56%) | 1 (5%) | 0 (0%) | 17 (19%) |
| No. trials with unexplained change | 5 (11%) | 3 (25%) | 1 (11%) | 5 (26%) | 1 (50%) | 15 (17%) |
| Covariates |  |  |  |  |  |  |
| No. trials with unexplained discrepancy | 9 (19%) | 6 (50%) | 4 (44%) | 4 (21%) | 0 (0%) | 23 (26%) |
| No. trials with unexplained addition | 3 (6%) | 3 (25%) | 1 (11%) | 0 (0%) | 0 (0%) | 7 (8%) |
| No. trials with unexplained change | 6 (13%) | 3 (25%) | 3 (33%) | 4 (21%) | 0 (0%) | 16 (18%) |
| Missing data |  |  |  |  |  |  |
| No. trials with unexplained discrepancy | 4 (9%) | 4 (33%) | 4 (44%) | 4 (21%) | 0 (0%) | 16 (18%) |
| No. trials with unexplained addition | 4 (9%) | 3 (25%) | 3 (33%) | 3 (16%) | 0 (0%) | 13 (15%) |
| No. trials with unexplained change | 0 (0%) | 1 (8%) | 1 (11%) | 1 (5%) | 0 (0%) | 3 (3%) |

^a^Included 5 trials with no discrepancies (4 pharma and 1 other) and trials 17 trials with all discrepancies explained (9 pharma, 2 surgical, 5 other, 1 multiple). ^b^Unclear if discrepancy occurred (n=11 including 8 pharma and 3 other), unclear if discrepancy explained (n=2 including 1 pharma and 1 other).

### **Appendix Table 9. Post-hoc subgroup analysis by availability of original analysis plan pre-results**

| **Unexplained discrepancies** | **OAP not available pre-results (n=43)** | **OAP available pre-results (n=46)** | **All**  **(n=89)** |
| --- | --- | --- | --- |
| None | 12 (28%) | 10 (22%) | 22^a^ (25%) |
| Yes | 20 (47%) | 34 (74%) | 54 (61%) |
| Unclear | 11 (26%) | 2 (4%) | 13^b^ (15%) |
| Total no. of analysis elements with unexplained discrepancy |  |  |  |
| 0* | 12 (28%) | 10 (22%) | 22 (25%) |
| 1 | 10 (33%) | 15 (35%) | 25 (28%) |
| 2 | 5 (17%) | 11 (26%) | 16 (18%) |
| 3 | 4 (13%) | 7 (16%) | 11 (12%) |
| 4 | 1 (3%) | 1 (2%) | 2 (2%) |
| Unclear | 11 (26%) | 2 (4%) | 13^b^ (15%) |
| Analysis population |  |  |  |
| No. trials with unexplained discrepancy | 10 (23%) | 18 (39%) | 28 (31%) |
| No. trials with unexplained addition | 7 (16%) | 9 (20%) | 16 (18%) |
| No. trials with unexplained change | 3 (7%) | 9 (20%) | 12 (13%) |
| Analysis model |  |  |  |
| No. trials with unexplained discrepancy | 10 (23%) | 21 (46%) | 31 (35%) |
| No. trials with unexplained addition | 6 (14%) | 11 (24%) | 17 (19%) |
| No. trials with unexplained change | 5 (12%) | 10 (22%) | 15 (17%) |
| Covariates |  |  |  |
| No. trials with unexplained discrepancy | 9 (21%) | 14 (30%) | 23 (26%) |
| No. trials with unexplained addition | 4 (9%) | 3 (7%) | 7 (8%) |
| No. trials with unexplained change | 5 (12%) | 11 (24%) | 16 (18%) |
| Missing data |  |  |  |
| No. trials with unexplained discrepancy | 7 (16%) | 9 (20%) | 16 (18%) |
| No. trials with unexplained addition | 5 (12%) | 8 (17%) | 13 (15%) |
| No. trials with unexplained change | 2 (5%) | 1 (2%) | 3 (3%) |

^a^Included 5 trials with no discrepancies (4 no OAP available pre-results, 1 OAP available pre-results) and 17 trials with all discrepancies explained (8 no OAP available pre-results, 9 OAP available pre-results). ^b^Unclear if discrepancy occurred (n=11 including 9 no OAP available pre-results and 2 OAP available pre-results), unclear if discrepancy explained (n=2 no OAP available pre-results).

### **Appendix Table 10. Post-hoc subgroup analysis by SAP availability**

| **Unexplained discrepancies** | **No SAP available (n=43)** | **SAP available (n=46)** | **All**  **(n=89)** |
| --- | --- | --- | --- |
| None | 6 (14%) | 16 (35%) | 22^a^ (25%) |
| Yes | 32 (74%) | 22 (48%) | 54 (61%) |
| Unclear | 5 (12%) | 8 (17%) | 13^b^ (15%) |
| Total no. of analysis elements with unexplained discrepancy |  |  |  |
| 0* | 6 (14%) | 16 (35%) | 22 (25%) |
| 1 | 10 (23%) | 15 (33%) | 25 (28%) |
| 2 | 12 (28%) | 4 (9%) | 16 (18%) |
| 3 | 9 (21%) | 2 (4%) | 11 (12%) |
| 4 | 1 (2%) | 1 (2%) | 2 (2%) |
| Unclear | 5 (12%) | 8 (17%) | 13^b^ (15%) |
| Analysis population |  |  |  |
| No. trials with unexplained discrepancy | 17 (40%) | 11 (24%) | 28 (31%) |
| No. trials with unexplained addition | 10 (23%) | 6 (13%) | 16 (18%) |
| No. trials with unexplained change | 7 (16%) | 5 (11%) | 12 (13%) |
| Analysis model |  |  |  |
| No. trials with unexplained discrepancy | 22 (51%) | 9 (20%) | 31 (35%) |
| No. trials with unexplained addition | 10 (23%) | 7 (15%) | 17 (19%) |
| No. trials with unexplained change | 12 (28%) | 3 (7%) | 15 (17%) |
| Covariates |  |  |  |
| No. trials with unexplained discrepancy | 15 (35%) | 8 (17%) | 23 (26%) |
| No. trials with unexplained addition | 4 (9%) | 3 (7%) | 7 (8%) |
| No. trials with unexplained change | 11 (26%) | 5 (11%) | 16 (18%) |
| Missing data |  |  |  |
| No. trials with unexplained discrepancy | 11 (26%) | 5 (11%) | 16 (18%) |
| No. trials with unexplained addition | 9 (21%) | 4 (9%) | 13 (15%) |
| No. trials with unexplained change | 2 (5%) | 1 (2%) | 3 (3%) |

^a^Included 5 trials with no discrepancies (3 no SAP available, 2 SAP available) and trials 17 trials with all discrepancies explained (3 no SAP available, 14 SAP available). ^b^Unclear if discrepancy occurred (n=11 including 5 no SAP available and 6 SAP available), unclear if discrepancy explained (n=2 with SAP available).

### **Appendix Table 11. P-values by main outcomes**

| **Outcome** | **N** | **Median P-value (IQR)** | **Min, Max** |
| --- | --- | --- | --- |
| All articles | 92 | 0.0065 (0.0010, 0.1900) | 0.0010, 0.1900 |
| No pre-specified analysis | 10 | 0.0002 (0.0001, 0.0029) | 0.0001, 0.0430 |
| Pre-specified analysis | 82 | 0.0112 (0.0010, 0.2200) | 0.0010, 0.2200 |
| If had pre-specified analysis: |  |  |  |
| Assuming unclear†=unexplained discrepancy |  |  |  |
| ≥ 1 unexplained discrepancy  change/addition | 60 | 0.013 (0.001, 0.305) | 0.001, 0.96 |
| No unexplained discrepancy | 22 | 0.0035 (0.001, 0.110) | 0.0001, 0.50 |
| No. of analysis elements with unexplained  discrepancy |  |  |  |
| 0 | 22 | 0.0035 (0.001, 0.11) | 0.0001, 0.5 |
| 1 | 32 | 0.0162 (0.001, 0.4) | 0.001, 0.96 |
| 2 | 16 | 0.013 (0.001, 0.1335) | 0.001, 0.81 |
| 3 | 9 | 0.0042 (0.0014, 0.31) | 0.001, 0.46 |
| 4 | 3 | 0.018 (0.01, 0.22) | 0.01, 0.22 |
| Assuming Unclear† =no unexplained discrepancy |  |  |  |
| ≥ 1 unexplained discrepancy  change/addition | 47 | 0.018 (0.002, 0.33) | 0.001, 0.96 |
| No unexplained discrepancy | 35 | 0.002 (0.001, 0.100) | 0.0001, 0.65 |
| No. of analysis elements with unexplained  discrepancy |  |  |  |
| 0 | 35 | 0.002 (0.001, 0.1) | 0.0001, 0.65 |
| 1 | 24 | 0.1 (0.0025, 0.52) | 0.001, 0.96 |
| 2 | 13 | 0.013 (0.001, 0.089) | 0.001, 0.81 |
| 3 | 8 | 0.0121 (0.0027, 0.345) | 0.001, 0.46 |
| 4 | 2 | 0.119 (0.018, 0.22) | 0.018, 0.22 |
| Exclude unclear† |  |  |  |
| ≥ 1 unexplained discrepancy  change/addition | 47 | 0.018 (0.002, 0.330) | 0.001, 0.96 |
| No unexplained discrepancy | 22 | 0.0035 (0.001, 0.11) | 0.0001, 0.5 |
| No. of analysis elements with unexplained  discrepancy  elements with  unexplained  change/addition |  |  |  |
| 0 | 22 | 0.0035 (0.001, 0.11) | 0.0001, 0.5 |
| 1 | 24 | 0.1 (0.0025, 0.52) | 0.001, 0.96 |
| 2 | 13 | 0.013 (0.001, 0.089) | 0.001, 0.81 |
| 3 | 8 | 0.0121 (0.0027, 0.345) | 0.001, 0.46 |
| 4 | 2 | 0.119 (0.018, 0.22) | 0.018, 0.22 |

†n=13 unclear whether ≥1 unexplained discrepancy occurred (n=11 where unclear whether discrepancy occurred and n=2 where discrepancy occurred but not clear whether explained.) For p-values reported as less than a number (e.g. <0.001), we analysed these at the stated value (i.e. for a p-value reported as <0.001 we included it in the analysis as 0.001).

### **Appendix Figure 1. Distribution of p-value for primary outcome by availability of pre-specified analysis approach**


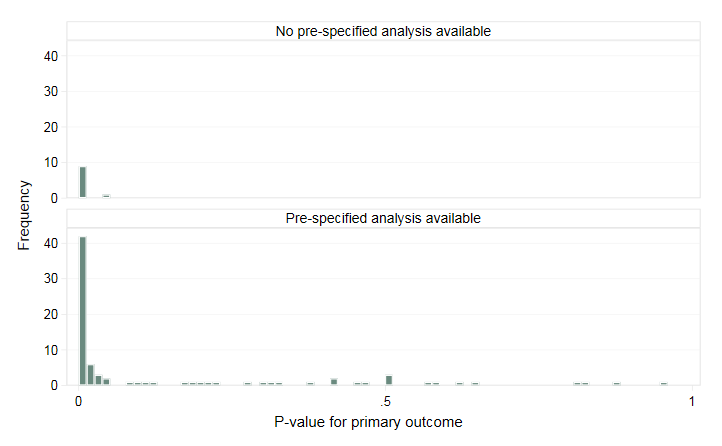


### **Appendix Figure 2. Distribution of p-value for primary outcome by unexplained discrepancies**

### **Appendix Figure 3. Distribution of p-value for primary outcome for p<0.15 by unexplained discrepancies**

## **Appendix 4: Statistical methods and results for analysis of trial outcomes**

**Statistical Methods**

We assessed whether availability of a pre-specified analysis approach or occurrence of unexplained discrepancies was associated with study results using linear regression analysis. We used the reported p-value for the primary outcome as a proxy for study results; we chose to use the p-value instead of the size of the estimated treatment effect because the p-value is a function of both the estimated treatment effect and its standard error, either of which may be affected. Further, different studies reported different treatment effect types (e.g. a difference in means for a continuous outcome vs. an odds ratio for a binary outcome) which can be difficult to compare. The log(p-value) was used as an outcome in linear regression models, where log denotes the natural logarithm. We evaluated the effect of each of the following three variables in separate models: (i) availability of a pre-specified analysis approach for the primary outcome (yes vs. no); (ii) any unexplained discrepancies (yes vs. no); and (iii) the number of analysis elements with an unexplained discrepancy (0-4, included as a continuous variable, assuming a linear association with outcome). Analyses (ii) and (iii) were restricted to the subset of trials with a pre-specified analysis approach. Each analysis included the following variables as potential confounders: journal, funding source (for-profit vs. not for-profit funding), type of intervention, and sample size (fitted as a continuous variable, using restricted cubic splines with three knots). For p-values reported as less than a number (e.g. <0.001), we analysed these at the stated value (i.e. for a p-value reported as <0.001 we included it in the analysis as 0.001).

For analyses (ii) and (iii) we made a change to our pre-planned analysis approach on how unclear discrepancies would be handled as we had not anticipated this issue. We did not pre-specify how ‘unclear’ discrepancies would be handled for analyses (ii) and (iii), as we had not anticipated this issue. We therefore made a post-hoc decision after data extraction was complete to classify unclear discrepancies as no unexplained discrepancy in the analysis; we performed two additional analyses where we classified unclear discrepancies as unexplained discrepancies, and by excluding articles with any unclear discrepancies from the analysis.

***Association between availability of pre-specified analysis approach or unexplained discrepancies and p-value***

Overall, 92 trials reported a p-value for the primary outcome. In 10 trials without a publicly available pre-specified analysis approach the median p-value was 0.0002 (IQR 0.0001 to 0.0029), compared to a median of 0.0112 (IQR 0.001 to 0.22) in 82 trials with a pre-specified approach (ratio [unavailable vs. available pre-specified approach] 0.02, 95% CI 0.0028 to 0.15, p<0.001).

In 60 trials with one or more unexplained discrepancies the median p-value was 0.013 (IQR 0.001 to 0.305), compared to a median of 0.0035 (IQR 0.001 to 0.110) in 22 trials with no unexplained discrepancies (ratio [≥1 vs. no unexplained discrepancies] 2.27, 95% CI 0.65-7.91, p=0.193).

### **Appendix Table 12. Associations with p-value for primary outcome**

| **Characteristic** | **N** | **Estimated ratio effect** | **95% confidence interval** | **p-value** |
| --- | --- | --- | --- | --- |
| No pre-specified analysis approach for primary outcome | 92 | 0.02 | 0.0028 to 0.15 | <0.0001 |
| Assuming unclear^a^=unexplained discrepancy |  |  |  |  |
| ≥ 1 unexplained  discrepancy (yes/no) | 82 | 2.27 | 0.65 to 7.91 | 0.193 |
| No. of analysis  elements with  unexplained  discrepancy | 82 | 1.09 | 0.62 to 1.89 | 0.768 |
| Assuming Unclear^a^ =no unexplained discrepancy |  |  |  |  |
| ≥ 1 unexplained  discrepancy (yes/no) | 82 | 2.23 | 0.65 to 7.70 | 0.202 |
| No. of analysis  elements with  unexplained  discrepancy | 82 | 1.15 | 0.64 to 2.08 | 0.632 |
| Exclude unclear^a^ |  |  |  |  |
| ≥ 1 unexplained  discrepancy (yes/no) | 69 | 2.33 | 0.61 to 8.89 | 0.211 |
| No. of analysis  elements with  unexplained  discrepancy | 69 | 1.16 | 0.63 to 2.16 | 0.626 |

^a^n=13 unclear whether ≥1 unexplained discrepancy occurred (n=11 where unclear whether discrepancy occurred and n=2 where discrepancy occurred but not clear whether explained.) For p-values reported as less than a number (e.g. <0.001), we analysed these at the stated value (i.e. for a p-value reported as <0.001 we included it in the analysis as 0.001).
